# Health-related quality of life, palliative care needs and 12-month survival among patients with end stage renal disease in Uganda: protocol for a mixed methods longitudinal study

**DOI:** 10.1186/s12882-020-02197-7

**Published:** 2020-12-07

**Authors:** Peace Bagasha, Mhoira Leng, Elly Katabira, Mila Petrova

**Affiliations:** 1 Department of Internal medicine, Makerere University College of Health Sciences, School of Medicine, P.O. Box 7072, Kampala, Uganda; 2 Makerere-Mulago Palliative Care Unit, Clinical Research Building, Mulago hospital site, P.O.Box 7072, Kampala, Uganda; 3 Cambridge Palliative and End of Life Care Research Group, Primary Care Unit, Department of Public Health and Primary Care, University of Cambridge; Cambridge Institute of Public Health, Forvie Site, Cambridge, CB2 0SR UK

**Keywords:** Quality of life [MeSH], Kidney failure, Chronic [MeSH], Palliative care [MeSH] renal Dialysis [MeSH], Developing countries [MeSH], Mixed methods, Resource limited setting, Low and middle income countries, LMIC

## Abstract

**Background:**

The prevalence of chronic kidney disease is on the rise globally and in sub-Saharan Africa. Due to its “silent” nature, many patients often present with advanced disease. At this point options for care are often limited to renal replacement therapies such as hemodialysis and kidney transplantation. In resource limited settings, these options are associated with catastrophic expenditures and increased household poverty levels. Early palliative care interventions, if shown to ensure comparable quality of life (QoL), can significantly mitigate this by focusing care on comfort, symptom control and QoL rather than primarily on prolonging survival.

****Method**s:**

A mixed methods longitudinal study, recruiting patients with End Stage Renal Disease (ESRD) on hemodialysis or conservative management and following them up over 12 months. The study aims are to: 1) measure and compare the health-related quality of life (HRQoL) scores of patients with ESRD receiving hemodialysis with those receiving conservative management, 2) measure and compare the palliative care needs and outcomes of patients in the two groups, 3) explore the impact of treatment modality and demographic, socio-economic and financial factors on QoL and palliative care needs and outcomes, 4) review patient survival over 12 months and 5) explore the patients’ lived experiences. The Kidney Disease Quality Of Life Short Form version 1.3 (KDQOL-SF) will be used to measure HRQoL; the African Palliative Care Association Palliative care Outcome Score (APCA POS) and the Palliative care Outcome Score for renal symptoms (POS-S Renal) will be used to assess palliative care needs and outcomes; and semi-structured in-depth interviews to explore the patients’ experiences of living with ESRD. Data collection will be carried out at 0, 3, 6, 9 and 12 months.

**Discussion:**

To the best of our knowledge, no similar study has been conducted in sub-Saharan Africa. This will be an important step towards raising awareness of patients’ need and preferences and the strengths and limitations of available health care services for ESRD in resource limited settings.

## Background

The global prevalence of Chronic Kidney Disease (CKD) is estimated at 8–16% [1–3], with this patient population being at the highest risk for progression to End Stage Renal Disease (ESRD). The prevalence of CKD in sub-Saharan Africa (SSA) at 12–23% [4–6] is higher than the global average of 13.4% [3]. This may be explained by the current burden of communicable diseases as well as the growing pandemic of non-communicable diseases such as diabetes and hypertension, which are the leading causes of CKD worldwide. Prevalence of CKD increases with age and as the aging population in low and middle income countries increases, prevalence of CKD is projected to increase further [3, 4, 6, 7].

CKD places significant demands on health systems in the form of medical consultations, medications, and renal replacement therapies (RRT) such as hemodialysis and kidney transplants. In addition to the lived experience of disease burden and direct health care costs, CKD patients and their carers have to face costs which often remain unacknowledged at the system level, such as of transportation, accommodation in greater proximity to centres of care, and time spent away from productive employment. A systematic review of the worldwide access to RRT in 2010 estimated that 2·6 million people were receiving such therapy, yet almost two to four times more, 4.9 to 9.7 million, were in need of it without having access to it. Those estimates were projected to mean that 2.3 million people might have died prematurely due to lack of access to RRT. Numbers continue to rise, with the largest treatment gaps occurring in low-income countries, particularly Asia (1·907 million people needing but not receiving RRT; conservative model) and Africa (432 000 people; conservative model) [8].

The World Health Organization (WHO) recognises the improvement/ minimization of deterioration of a patient’s quality of life as a key outcome of any successful disease management and ESRD is no exception [9]. ESRD has been shown to be associated with poor Health-Related Quality of Life (HRQoL) in both developing and developed countries, for instance Kenya, Guinea, India and the United Kingdom [8–11]. Factors significantly associated with poor HRQoL included: older age, higher symptom burden and comorbidities, more physical pain and more physical and social limitations [9]. In Malawi low household income (< $4000 per year) was associated with lower scores on the mental health component and low physical health scores were found to discourage many patients from seeking treatment, particularly if they were the bread winners in their families [10].

The role of palliative care, an approach that aims to improve quality of life, in ESRD has been recognized and developed in USA, UK, Canada, Australia and most developed countries [11–14]. It involves helping patients to cope with their illness and choose care pathways (dialysis vs. conservative care, withholding dialysis, making advance care directives), and provides a multidisciplinary holistic approach to care at the end of life [11, 15, 16]. Patients with ESRD, irrespective of their management modality, have significant morbidity and mortality, with one study showing an average of 10.5 symptoms in the severely ill [15]. These patients have been shown to benefit significantly from palliative care services [11, 17, 18]. In practice, however, palliative care is offered to patients on conservative management more often than it is offered to patients on hemodialysis. Also, there are no well-established palliative care pathways for such patients even in developed settings.

There is a scarcity of evidence on palliative care in ESRD from developing countries and a significant variation in the models of palliative care delivery in ESRD in developed countries.. This has made it difficult to formulate guidelines for palliative care in ESRD in developing countries. This study therefore aims to bridge this gap by contributing to the limited body of knowledge on palliative care and HRQoL in ESRD patients in developing countries.

## Methods/study design

This study is a doctoral project of the first author (PB) funded by the Training Health Researchers into Vocational Excellence (THRiVE) consortium in Uganda. It is a mixed methods longitudinal study, with a 1-year follow up. Quantitative (Part 1) and Qualitative (Part 2) arms of the study will take place concurrently, both in terms of data collection and analysis.

### Aims and objectives

The overall aim of the study is to improve our understanding of quality of life amongst patients with ESRD receiving care in a resource limited setting – Mulago National Referral Hospital in Uganda, and the implications this has for the provision of palliative care in addition to standard nephrology care in resource limited settings.

The objectives of the quantitative arm of the study are:

1. To measure the HRQoL scores of a sample of patients with ESRD receiving care at Mulago National Referral Hospital;
2. To compare the HRQoL scores of a sub-sample of the above patients who are receiving hemodialysis to the scores of a sub-sample of patients who are on conservative management;
3. To elicit and compare the factors that influence the HRQoL of those two sub-samples of patients;
4. To calculate the 12-month mortality rate of the full sample of patients.

The objective of the qualitative arm of the study is:

1. To explore the lived experience of ESRD of the recruited patients.

Longitudinal follow-up will be for 12 months, with quantitative reviews every 3 months and repeat qualitative interviews every 6 months. In following up these patients we are assessing changes to their quality of life, management modalities they are utilizing and number of admissions as well as place of death for patients who die during the course of follow up. Follow up will not include blood tests to examine serum biochemical changes at every interaction.

### Study site

Uganda is a sub-Saharan country located in East Africa with an estimated population of 40 million [19]. Mulago Hospital is the largest health care facility in the country with a bed capacity of 1500. It is the main national referral hospital, meaning that the majority of the patients seen have been referred from lower tier health care facilities due to the complexity of the patient’s illness. All adult medical inpatient and outpatient services are currently seen at the Kirrudu site, where all the data collection will take place other than in cases where interviews are conducted, as per the patient’s preference for example in their home .

Adult patients admitted to the medical wards come in through the medical emergency ward or the outpatient clinics. Patients with confirmed or suspected kidney disease are admitted to the nephrology ward which, on average, provides care for 15–30 patients daily. The outpatients’ clinic runs every Tuesday, with 5–10 new and 20–25 follow-up patients per week. The hemodialysis unit, housed in the same building, operates 15 to 20 functional dialysis machines and runs two to three patient shifts daily, 6 days a week. It has 150–200 active hemodialysis patients at any one point. The Unit being a government run public facility requires patients to pay a highly subsided cost for dialysis for only two sessions per week but they have to pay the full amount if they have to undergo a third session. From anecdotal evidence patients with CKD in this setting are started on hemodialysis by the attending nephrologist when they develop features of the Uremic syndrome, volume overload, metabolic acidosis or hyperkalaemia all of which are refractory to treatment. A decision to initiate or withhold dialysis involves all relevant parties including the carers, patient and healthcare workers agreeing together on a course of action. Often the main factor leading to no dialysis and therefore conservative management is the lack of funds to pay for catheterization and the hemodialysis procedure not because they are very sick. Occasionally, especially in patients aged over 75 years, extensive co-morbidities may be a factor, but this is not very common because the average population age in our setting is 45.9 years.

### Study approvals

Ethical approval was provided by the School of Medicine Research and Ethics Committee of Makerere University (#REC REF 2018–005). Administrative clearance to carry out this study at Mulago National Referral Hospital was given by the Hospital Administration following review and approval by the Mulago Hospital Research and Ethics Review Committee (MHREC 1543). The Uganda National Council for Science and Technology (UNCST) also reviewed and approved the study (HS 2573).

### Study population

To be eligible for participation in the study, patients must be aged 18 years and above with documented evidence of ESRD defined as CKD stage V (estimated glomerular filtration rate of 15mls/min/1.73m^2^ or less calculated using Cockcroft-Gault Formula). They should be admitted on the nephrology inpatient ward or to be attending the outpatient nephrology or hemodialysis clinics. They should have decision making capacity to enable them to give informed consent to take part in the study, as well as adequate cognitive and linguistic competence and levels of physical functioning to allow them to complete a questionnaire and participate in an interview without undue strain.

Patients will be excluded if they do not have access to a mobile telephone: this may be a personal mobile phone or one belonging to a trusted friend or family member who can easily access the patient and whose knowledge of the patient’s condition and study participation is fully acceptable to the patient.

Patients with acute kidney injury defined as an elevated serum creatinine for a period of less than 3 months as well as patients who do not meet the above criteria about decision making capacity, cognitive and linguistic competence and levels of physical functioning will also be excluded.

### Participant recruitment

Patients meeting the inclusion criteria will be recruited through non-probability consecutive sampling from the nephrology inpatient ward, the general nephrology outpatient clinic (once weekly - Tuesday) and the outpatient hemodialysis unit (Monday-Saturday). We chose this recruitment design because of the small overall patient numbers and anecdotal evidence of high morbidity and mortality amongst them. There is a significant probability for a large number of unsuccessful recruitment attempts due to the severity of the illness with which patients present at the hospital. Significant drop-out rates are also expected due to the high likelihood of patient deterioration post-recruitment or death during the study period.

The recruitment will be conducted by two research assistants, both of whom are health care assistants with diploma level medical training. They will receive training in patient identification and recruitment, data collection methods and the ethical conduct of research from the doctoral candidate and primary investigator (PB). The research assistants, upon identifying an eligible patient, will introduce themselves and explain the purpose and processes of the study. They will take extra care to clarify that the patient’s decision will in no way influence the health care they receive and that, even if they decide to take part, they are free to withdraw at any time. The patient will be encouraged to ask questions about the study and to take time to consider whether they might be interested in participating. If the patient expresses an interest to join the study, they will be asked to sign a consent form and given an information sheet to keep.

### Quantitative arm

#### Sample size estimation

The sample size needed to compare the mean HRQoL values for the 2 patient groups (on hemodialysis vs. conservative management) was estimated as follows:

#### Calculating sample size to achieve adequate power; comparison of two means

$$ \mathbf{n}=\left[\ {\left(\ {\mathbf{z}}_{\mathbf{1}}+{\mathbf{z}}_{\mathbf{2}}\right)}^{\mathbf{2}}\mathbf{x}\ \mathbf{2}\left(\ {\mathbf{s}}^{\mathbf{2}}\right)\ \right]\kern0.75em /\kern0.5em \left({\boldsymbol{\upmu}}_{\mathbf{2}}-{\boldsymbol{\upmu}}_{\mathbf{1}}\right)\mathbf{2} $$

**WHERE.**

n = sample size per group.

z_1_ = 1.96 for error of 5% (95% confidence level).

z_2_ = 1. 64 for 95% power.

= 1.28 for 90% power.

= 0.84 for 80% power.

s = standard deviation for of the outcome in control group.

μ_2_ - μ_1_ = minimum meaningful difference between means of the intervention and control group [20].

The calculation used z_2_ at the 0.84 level for 80% power, meaning we expect a sensitivity of 80% from our study findings. We used as a reference for the calculations the study of HRQoL in kidney disease by Manavalan et al. conducted in another setting with likely resource limitations, namely South India [21]. The study used the Kidney Disease Quality Of Life-Short Form (KDQOL-SF) Version 1.3, which is also one of the tools to be used in this study (see Data collection and Table 1 below). Patients had chronic kidney disease, were from an area of predominantly low social and economic status, and were seen in a tertiary referral center. In view of such similarities, the values of S = 13.4 (standard deviation for the control group, taken to be conservative management group) and μ_**2**_ **-** μ_1_ = 5.08 (the minimum meaningful difference between means of the intervention and control group, observed in the ‘Effects of kidney disease’ item under the kidney disease specific domain) were taken from the Manavalan et el. study [9].

$$ \mathrm{N}=\left({\left(1.96+0.84\right)}^2\ast 2\ast {(13.4)}^2\right)/{(5.08)}^2=109\ \mathrm{participants}\ \mathrm{per}\ \mathrm{group} $$

$$ \mathrm{Total}\ \mathrm{participants}=109\ast 2=218 $$

**Table 1:** Data collection tools

• Kidney Disease Quality Of Life-Short Form (KDQOL-SF) Version 1.3	The KDQOL-SF questionnaire (version 1.3) is an internationally validated instrument for assessing the HRQoL of ESRD patients (41) and hemodialysis patients (42, 43). It consists of measures of general health and measures specific to ESRD organized in three domains: - Physical health Component Summary (PCS), 21 items; - Mental health Component Summary (MCS), 15 items; - Kidney Disease Component Summary (KDCS), 44 items. A total score and domain-specific PCS, MCS and KDCS will be calculated for each patient and a mean score calculated for each of the sub-samples. Total scores above 50 (max 100) are considered to represent good levels of quality of life.
• Socio-demographic data	Demographic data: age, sex and marital status Socio-economic data: educational level and employment status Financial data: (approximate) total yearly household income and expenditures on health care.
• The African Palliative Care Association Palliative care Outcome Scale (APCA-POS)	The APCA-POS, validated in Uganda, assesses patient palliative care needs and outcomes of care [22]. It contains 10 items, seven for the patient only and three for the family. The following aspects of holistic end of life care are explored: - Physical (pain and symptoms) - Psychological (patient and family worries) - Existential / Spiritual (worthiness of life, feeling at peace) - Social (confidence in caring for the patient). Each item is scored on a scale of 0 to 5.
• The Renal symptoms Palliative care Outcome Score (POS-S Renal).	The POS-S Renal assesses palliative care needs and outcomes specifically in patients with kidney disease [23]. It assesses how a patient feels across a range of symptoms specific to kidney failure (scale of 0 to 4). It also elicits which symptom a patient feels has affected them the most and which has improved the most.

Delays in presentation to tertiary care facilities often result in advanced disease at presentation and are related to poor survival rates [24, 25]. Based on such evidence and observations of the PI and three colleagues (two senior hemodialysis nurses and a nephrologist), all of whom members of the Mulago Hospital nephrology team, we anticipated a high level of attrition, which we set at 30%.

Adjustment for attrition: *N*^*1*^ *= N* / (1-q), [20].

where N^1^ is adjusted sample size, N is unadjusted sample size and q is the attrition rate

$$ {\mathrm{N}}^1=218/\left(1-0.3\right)=311 $$

We also adjusted for unequal group sizes because fewer patients, due to cost and accessibility challenges, are able to undergo hemodialysis [26].

Adjustment for unequal group sizes: *N*^*1*^ *= N* (1 + k) ^2^ / 4 k [20].

N^1^ is adjusted sample size, N is unadjusted sample size, k is the ratio of the 2 groups = 1: 2

$$ {\mathrm{N}}^1=\left(311\ {\left(1+0.5\right)}^2\right)/4\ast 0.5=350 $$

Thus, 117 (350/3) patients will be allocated to the hemodialysis group and 233 (350* 2/3) to the conservative management group.

For each participant, data will be collected over a period of 12 months or less if their condition deteriorates significantly or if they die.

### Data collection

The data collection questionnaire will consist of the tools described in Table 1.

The KDQOL-SF was developed by the RAND Corporation and is a public document available for use by any interested parties [27]. It is among the mostly widely used QoL instruments specific to end stage kidney disease and has been validated and used extensively in both developed and developing countries [10, 21, 28, 29]. As part of the current study, the tool has been translated and adapted to reflect the specificities of Ugandan culture.

The process of translation followed the guidelines provided by RAND Health Care [30] (Appendix B). The translation was into one of the local languages, Luganda, the most commonly spoken language in the central region of Uganda, where Mulago Hospital is located. A team of three native Luganda speakers worked together to translate the instrument into Luganda. Back translation into English was then done by a fourth native Luganda speaker, who is also a linguist. The tool was than tested to assess face and content validity in one focus group discussion involving nine health care workers and in five individual interviews. Culturally, the tool was adapted by substituting activities which are uncommon in the local setting, such as vacuuming or bowling, with far more typical ones, such as sweeping or mopping the house.

The African Palliative Care Association – Palliative care Outcome Scale (APCA POS), validated in Uganda [22], contains 10 items, addressing the physical and psychological symptoms, spiritual, practical and emotional concerns, and psychosocial needs of the patient and family. Answers to all questions are scored using Likert scales from 0 to 5, with numerical and descriptive labels. Questions 1–7 are directed at patients; questions 8–10 are directed at family informal caregivers and include a ‘Not applicable’ option for use when the patient does not have an informal carer. It is staff-completed, owing to varying levels of patient and family literacy. Respondents indicate their answers either verbally or using a hand scale (0 = closed fist, 5 = all fingers open). Like in the KDQOL-SF, the scoring of some items is reversed, meaning that best status can be represented by both high and low scores. This ensures that administration and response formulation for each individual item are conducted with due care and attention.

The Palliative care Outcome Scale - Renal has been extensively used to assess the palliative care needs and outcomes specifically in patients with kidney disease [16, 23]. It assesses how a patient feels across a range of symptoms specific to kidney failure (scale of 0 to 4), including a *Not at all* option for patients not experiencing a particular symptom. In addition, it elicits which symptom a patient feels has affected them the most and which has improved the most.

Together, the above three instruments will provide a multi-factorial quantitative perspective on the patients’ quality of life. Associations will also be explored between scores on each of the three questionnaires and the hypothesized predictors of HRQoL, including management modality and the parameters included in the set of sociodemographic data to be collected (see Table 1).

A pilot study involving 38 study participants was carried out in May 2018. It showed that patients preferred to have the questionnaire administered to them by the research assistants. The questionnaire will therefore be printed on paper and the two research assistants will administer it to the eligible patients at the various study sites: in the waiting areas; in the hemodialysis unit while a patient is undergoing dialysis; or at a patient’s bedside in the nephrology unit. The research assistants have been trained to maintain consistency in the administration of the questionnaire, to help the patient understand the meaning of the questions and offer support without influencing their responses. The PI has observed the research assistants administering the questionnaire during pilot data collection. Meetings were also held to enable further standardization of the process, e.g. to ensure that the intended meanings of questions are uniformly understood. Based on the pilot, it is expected that the questionnaire will take about 30 min to administer. More time may be allowed for breaks depending on the participants’ needs. We anticipate administering 10 questionnaires per day. This will be done at 0, 3, 6, 9 and 12 months (see Table 2).

**Table 2:** Schedule of assessments

Assessment	Screening visit Baseline	Visit 1 3 months	Visit 2 6 months	Visit 3 9 months	Visit 4 12 months
Quantitative arm					
Recruitment relative to inclusion/exclusion criteria	X				
Informed consent	X	X	X	X	X
Demographic data	X				
Management modality patient is on	X	X	X	X	X
KDQOL	X	X	X	X	X
APCA POS	X	X	X	X	X
POS-S Renal	X	X	X	X	X
Qualitative arm					
Patient interview	X		X		X

Two phone calls will be made in between patient reviews to ensure close contact and reduce loss to follow up. The first will be made at 2 months after a review. The second will be made 1 week before the next review to agree time and venue which are most convenient to the patient.

### Qualitative arm

The goal of the qualitative aspect of the study is to explore the multidimensional lived experience of a sub-sample of the recruited patients at three time points: 0, 6 and 12 months. Dimensions of interest will be the physical, psychological, social, spiritual and pragmatic/ daily living aspects of the patients’ experience and the factors that influence them. The knowledge gained from the qualitative arm will be used to complement, augment, explain and potentially challenge evidence obtained from the quantitative arm.

Data will be collected through semi-structured interviews (see Table 3 for schedule). The interviews will aim to cover six broad thematic areas, while giving as much opportunity to participants to lead the interview as possible. The probing questions will be used mostly if the interviewee needs clarification of the initial broad question for a theme. The overall goal will be to give space to the interviewee to structure the conversation and prioritize issues to talk about than to cover the variety of subtopics within a theme, as articulated in the probes.

**Table 3:** Semi-structured interviews guide

**Introductory remarks:**	
**• Transitional and ice-breaking comments and questions: follow situational cues to help the patient feel comfortable and establish a connection, e.g. ask about their trip to the hospital, their appointment today, etc.**	
**• Thank the interviewee again for agreeing to talk to you.**	
**• Confirm that their participation is entirely voluntary and that they can withdraw at any point without giving an explanation and without any consequences for the health care they receive.**	
**• Ask them to tell you if at any point they feel tired and want to stop to take a break.**	
**• Tell them that there are no right or wrong answers and you want to understand what they think and feel, that it is about their experiences and it doesn’t matter what anyone else tells them is right, true, good …, even if it is the doctor [add another authority – e.g. husband, mother, etc. if you have indications that they may have a significant influence on the patient]. If appropriate, can make a joke adding the President or somebody famous to that list of authorities.**	
**• Try to use as much of the language they are using – e.g. the way they call ‘End Stage Renal Disease’.**	
**1. You and your illness**	
**Core question:** Could you tell me about your kidney disease? For instance, how and when you developed it and if it has changed your life or not?	
*Possible probes if interviewee asks for further guidance [though first try to paraphrase and reassure that any way they understand it is good] or if their answer has been brief [again, first try to leave open – Anything else?]:*	
*-*When were you told that you have kidney disease?	
-Where was that?	
-What made you go to the doctor/ hospital?	
-What did you know about kidney disease at the time?	
-What do you think might have caused your kidney disease?	
-What treatment are you currently receiving?	
-Has kidney disease changed your life in some big ways?	
-What do you feel is your biggest challenge about living with it?	
**2. Physical Health**	
**Core question:** Can you tell me how you’ve been feeling physically over the past 6 months? What do you feel in your body?	
*Possible probes if interviewee asks for further guidance [though first try to paraphrase and reassure that any way they understand it is good] or if their answer has been brief [again, first try to leave open – Anything else?]:*	
-What physical ailments have you been having?	
-Which ones have disturbed you the most? In what ways?	
-Is the health care you are receiving helping enough with those ailments? Can you tell me a bit more? What is helping? What is not helping? What is good about it? What is not that good?	
-How many pills do you take in a day? How does it feel?	
-Are they too many? Difficult to swallow? Expensive to buy?	
-Do you feel they help enough? Or do they help for some things but make others worse?	
-Do you sometimes skip some doses?	
-How often do you come to the hospital?	
-How far do you live? How far is it for you to travel? Does it make it difficult to come?	
-What do you think about the care you are getting here at the hospital?	
-Can you give me some examples of both when it was good and bad, or not so good?	
**3. Daily lif**	
**Core question:** How has kidney disease changed what you can do in your everyday life? The normal things that you do from when you wake up in the morning to when you go to sleep at night?	
*Possible probes [again, use only after first opening space for the unguided understanding of the interviewee]:*	
***Eating and drinking***	
*-*How is your appetite?	
*-*Did you change the food you are eating? How are you finding the changes?	
-Do you miss some of the things you shouldn’t eat? How do you like the ‘new’ foods?	
-Is it difficult to find some of those foods?	
-What about the things you are drinking?	
***Movement, daily tasks and work***	
-Are there things that are more difficult to do because you feel weaker?	
-What about daily tasks and self-care? E.g. bathing, eating, turning in bed, transferring from bed to chair? What about vigorous activity, like fetching water?	
-What about working to earn money?	
-Going out of the house and visiting friends and family?	
***Sleep***	
-Do you have trouble sleeping?	
-What makes it worse? What makes it better?	
-What do you think about when you can’t fall asleep?	
-How do you feel if you haven’t slept well?	
***Family***	
-Who in your family knows about your illness? How much have you told them?	
-How did the conversation go?	
-What were their reactions?	
-How are they supporting you in living with the illness and taking care of your health?	
-In what ways are they helping? E.g. helping at home, visiting you in the hospital, looking after you in the hospital, bringing you food, paying some of your bills … .	
-My next questions may feel too personal, remember it’s entirely up to you how much or little (or not at all) you say, but how has the illness affected your relationship with your spouse or partner? Or if you are not in a relationship, your plans for one?	
- Has your illness affected your sexual relations?	
-Going back to the support you are getting from your family as a whole, can you give me some examples of when it felt good and when it didn’t?	
***Money***	
-Do you have a job or some other steady source of income?	
-How much money do you spend on your health care in a month? Medication, consultation fees for doctors, transport, food while in the hospital, rent, etc.?	
-How much of your monthly income is that?	
-How do you manage those expenditures?	
-Have you needed to borrow money?	
-Does spending on your health care mean others in the family can’t get what they need or have to wait for it longer? What do they say? How do you feel? [*Might need to close topic and move on with a compassionate comment that it’s a big and difficult topic.*]	
**4. Psychological Health**	
**Core question:** How has kidney disease affected how you feel? About yourself, about life in general? Has your usual mood changed?	
*Possible probes [again, use only after first opening space for their unguided understanding]:*	
*-* Do you feel that this disease is stressing you out? In what ways?	
-Do you feel like a burden to your family? Can you tell me a bit more?	
-Do you worry about your future? What do you most worry about?	
-Has this changed how you plan things?	
-Do you ever think about a time when you are no longer in this world?	
-If yes, what are your thoughts on this?	
-Have you made a will or discussed it with anyone?	
-If you don’t want to think about a future when you are no longer in the world, can you tell me very briefly why, only so that I may understand, not because I want to make you think about it.	
-Has your illness brought about anything positive? Do you sometimes feel good about it?	
**5. Social Health**	
**Core question:** Can you tell me how your illness has affected your relationships with other people? We spoke of your immediate family (and can come back to it), but perhaps we can talk more about your friends, neighbours, workmates, maybe other patients or even strangers?	
*Possible probes [again, use only after first opening space for their unguided understanding]:*	
-Do people other than your family help you in living with your illness or taking care of your health? E.g. neighbours, friends, workmates?	
-How are they trying to help you?	
-How do you feel about that? In what ways is it good? In what ways it isn’t?	
-Have you lost touch with some people because of your illness? How did it happen?	
-Do some organizations, such as NGOs, provide support? In what ways?	
-Do you sometimes feel lonely? Or too different from others?	
**6. Spiritual Health**	
**Core question:** It may feel a very big, serious question, and it’s entirely up to you if you want to think and talk about such things, but can you tell me what helps you cope with your illness or what gives you strength when facing a difficult illness such as the one you are going through?	
*Possible probes [again, use only after first opening space for their unguided understanding]:*	
-Do you believe in God or a higher power?	
-How has your illness affected your faith and your sense that life has a meaning?	
-Has your faith helped you accept and live a good life in spite of your illness? Can you tell me of such times?	
-Or has the opposite happened, you started losing faith because of your disease? Can you tell me of such times?	
-What gives you hope? Can you tell me of times when you had high hopes about your health?	
-What makes you lose hope about your health? Can you tell me of situations when that’s happened?	
-Are there any religious practices which help you cope with your illness?	
**7. Wrapping up**	
• Thank you for your time and contribution to this study, we are just wrapping up now. I would like to know, given what you have been through so far, what advice would you give to a fellow patient?	
• What about health care providers? How can they help patients better?	
• How about the Government and Ministry of Health? How can they contribute to improving your health as a kidney disease patient?	
• What information would you like to have about your health in general and kidney disease?	
• Is there anything else you’d like to tell me?	
• Is there anything else you’d like to ask me?	
**Thank you so much for your time and participation.**	

The same schedule will be used to guide the interviews at the three time points. After the original interview, the goal will be to achieve greater depth of understanding; to capture changes over time, as the patient’s experience with the illness grows; and to clarify points that have remained opaque during previous interviews. Before the 6- and 12-month interviews, the participant’s latest transcript will be reviewed to identify potential areas of focus for the upcoming interview.

### Patient sampling and recruitment

Interviewees will be recruited from amongst patients who have already agreed to participate in part 1 of the study, by asking them whether they would like to participate in an interview too or only respond to the questionnaire. Selected participants will have to be physically and cognitively able to participate in a 30–45 min in-depth interview and to have consented to the interview and the audio recording. We will use purposive maximum variation sampling, with variation based on age, sex, duration of illness, duration on treatment modality, estimated glomerular filtration rate at baseline, other morbidity, and HRQoL scores at baseline.

Once identified as fitting the above criteria, participants will be approached again by the research assistant who had administered the quantitative questionnaire and asked for a time and place convenient to interview them.

### Sample size estimation

The final sample size will be determined by the point at which we achieve data saturation, i.e. no new information or theme is identified during progressive interviews [31]. Based on comparable research, we estimate that data saturation will be achieved after 25–30 maximum variation patient interviews [32].

### Data collection

The interviews will be conducted face-to-face by the principle investigator assisted by one research assistant with training in qualitative interviews.

Field notes will be taken during and after the interviews to provide context for the data analysis and inform subsequent interviews. Interviews will be conducted in Luganda, English or Runyankole, depending on patient preference. The interviews will be audio-recorded, with patient permission. Interviews conducted in English will be transcribed verbatim. Interviews in the local dialects will be translated during transcription by study administrative staff highly competent in both the native language and English. These translations will then be checked by a native speaker of each language.

We expect to recruit 1–2 interviewees a week. Repeat interviews will be conducted at 6 and 12 months from the first interview.

### Data management and storage

Questionnaire data will be entered into EpiData Ver. 3.1 [33] after data cleaning. Interviews will be audio-recorded on a password-protected recorder, downloaded onto a password-protected laptop and deleted from the recorder at the end of each day. Printouts of anonymized interview transcripts will be stored securely in a locked cupboard. Physical data will be safely destroyed 5 years after the end of the study.

### Analysis plan

#### Quantitative analysis

Quantitative data will be analysed in STATA version 12.0 [34].

#### Primary outcomes

For objective one; overall scores will be calculated for each participant for the KDQOL-SF 1.3 using the manual for use and scoring provided by Hays et al. [27]. Individual scores will then be computed into means and then categorised into high and low scores (respectively > and < 50/100). Mean scores for each of the three domains will also be calculated and categorised (physical, mental and kidney disease specific, respectively > 50 and < 50% of the sub-scale maximum score; decision on treating mid-points will be taken in light of their frequency).

For objectives two and three; participants will be divided into the HD and non-HD groups based on the management they are receiving at the time of assessment and scores categorised into these groups. Exploratory analyses will be used to compare the mean at 3 months between categories of categorical variables (such as gender, marital status, comorbidity) using ANCOVA adjusting for baseline scores. The association between KDQOL-SF scores and other continuous variables will be investigated using simple linear regression with KDQOL-SF scores at 0 months as the dependent variable. Multiple linear regressions will be used to explore the relationship between KDQOL-SF, APCA POS and POS-S Renal scores on the one hand and, on the other, management modality, age, sex, marital status, educational level, employment status, total yearly household income and expenditures on health care. Statistical significance will be assumed for *P*-values < 0.05. Biases due to repeated measurements will be addressed during data collection by consistency and training of research assistants but also during analysis by using Generalized estimated equations (GEEs) and as required mixed –effects models.

For objective four: Descriptive summaries of the patient socio-demographic question-naire data at 0,3,6,9 and 12 months will be tabulated and presented graphically where appropriate. The number (%) of deaths at 3, 6, 9 and 12 months will be tabulated. Time to death data will be investigated using Kaplan Meier curves. The log rank test statistic will be used to compare categorical variables and Cox proportional hazards model for continuous variables where appropriate.

#### Qualitative analysis

Qualitative data will be analysed using NVivo 12 plus [35]. Interview transcripts and field notes will be read and reread several times with the intent of finding patterns and relationships, revealing deeper meanings and understanding participants’ stories. First cycle codes will be developed utilizing principles of open coding. Codes will describe sections of texts of various length forming a thematic unit. A second cycle of coding will then follow, involving constant comparison between new and existing codes and the interview excerpts which underpin them. This will lead to the formation of higher level categories and finally broad themes. Issues emerging from each interview will contribute to the questions asked alongside the existing interview guide at subsequent interviews until data saturation is reached [36].

Four researchers are involved in the coding of qualitative data. Two of these have PhDs and between them over five publications from qualitative studies they have been involved in. One is a PhD candidate who has have training in qualitative methodology and who will be doing the bulk of the work under the supervision of the more senior colleagues. The fourth is a palliative care nurse with over 10 years experience in patient care and qualitative data collection and analysis. All the four have reviewed at least two transcriptions and agreed on the codes developed.

#### Ethical considerations

To obtain informed consent a detailed explanation of the study aims, procedures, benefits and potential harm, and the intension to disseminate information gathered through publication and at local and international meetings of all relevant stakeholders, will be provided to the patients. Patients will be assured of their freedom to withdraw from the study whenever they wish.

To maintain patient confidentiality, each study participant will be assigned a patient identification code. Only the patient identification code will be used in the final data base. Each interview participant will be assigned a pseudonym in the transcripts. The key linking patient identification codes with patient names and/or other identifying data (e.g. for the purposes of checking mortality data) will be accessible only to the PI on a password protected computer.

We anticipate the following ethical dilemmas to arise and intend to use the following approaches to handle them:

a Choice of treatment (dialysis vs. conservative management), especially as kidney function declines: Patients will be fully counselled about their options and their decision will not be influenced by the study.
b Availability of specialist care and appropriate referrals not being possible for patients, especially those with financial limitations: This is an ethical dilemma which the team experiences on a regular basis. It has been one of the key motivations for conducting the study and collecting evidence that compares quality of life and outcomes for patients on hemodialysis and conservative management (with the latter demanding less specialist involvement and much less financial resource on the part of patients and families).
c Patients whose condition deteriorates as the study progress: We will ask for informed consent from the patient at each visit. Baseline consent will be both verbal and written and only verbal at subsequent interactions. Patients will be assured at all times that they can withdraw from the study at any point if they so wish.

#### Patient switch over

The study does not involve switching over of patients and this remains a decision of the hospital care teams. However, it is likely that some patients will choose to have their treatment changed during the course of the study. The data of patients who switch over from one arm to another will be analysed based on the arm they were in at the time of data collection. If these patients form a large enough group, further, more focused analysis will also be conducted.

## Discussion

Global standards of care for patients with chronic kidney disease aim to modify disease progression and holistically improve quality of life, including through the early integration of palliative care. High quality and multimodal evidence around quality of life in this patient group can help identify areas of greatest need and potential for improvements of care provision as well as serve as a baseline against which to evaluate improvement initiatives.

This study will utilize a mixed methods longitudinal design to explore the quality of life, palliative care needs and outcomes, lived experience and mortality of patients receiving treatment for ESRD in a resource limited setting. It will compare patients undergoing conservative management and hemodialysis management following them up for 12 months. It will generate local evidence that is, in many respects, unique for Uganda. To our knowledge, this is the first study which utilizes a mixed methods approach to explore HRQoL for patients in Uganda. It is also the first to collect qualitative data on chronic kidney disease patients in Uganda and on their palliative care needs and outcomes. Furthermore, it is the first study in the country to follow up a cohort of kidney disease patients to assess their 12-month survival.

Such evidence can be used to inform the development of interventions and support infrastructure for patients and their informal carers, training for health care workers, and information briefs for policy makers, as part of synergistic efforts to increase the quality of life of patients with End Stage Kidney Disease.

A main limitation of this study is the use of convenience sampling and one site for data collection. This will constrain the generalizability of findings. However, the study site is the largest renal center in Uganda, which receives referrals from all regions of the country, including three neighboring countries. A set of additional limitations concerns issues around languages and mobile phone access. The study questionnaire has been translated in only one of the local languages, be it the most widely spoken in the region. A proportion of the in-depth interviews will also be translated from local languages into English. While translations will be carried out by bilingual translators for each of the local languages to ensure the highest possible quality, there is a possibility that subtle differences and nuance, arising from differences in both the culture and language, are lost. Importantly, the study PI is a core member of the hospital nephrology team and much of the data collection is conducted in spaces where patients are receiving care. In spite of the careful explanations given by the research team, patients may still refrain from voicing concerns about the care they are receiving. Participants may also exhibit the Hawthorne effect, whereby the very participation in the study, with the significant attention and opportunities for sharing their experiences they are given, improves their overall experience of ESRD. Concerning mobile phone access, surveys by the National Information and Technology Authority (NITA) have demonstrated that 70.9% of individuals surveyed (2400 individuals sampled from all regions in the country) owned a mobile phone [37]. With no better options available for tracing and following participants we considered this to be an acceptable level of coverage. To minimize this limitation we included participants who did not own mobile phones but they had to have contacts of close relatives or friends with phone contacts and therefore enable us to get in touch with them.

While such limitations need to be taken seriously in interpreting the resulting evidence and improved upon in further research, this is, nonetheless, a unique study for End Stage Renal Disease patients in Uganda with important implications for the health care they receive. It is also an important step to raise awareness of the strengths and limitations of health care services in the country for what is, currently, its 5th most common cause of death.

## Data Availability

To maintain patient confidentiality, each study participant will be assigned a patient identification code. Only the patient identification code will be used in the final data base. Each interview participant will be assigned a pseudonym in the transcripts. The key linking patient identification codes with patient names and/or other identifying data (e.g. for the purposes of checking mortality data) will be accessible only to the PI on a password protected computer. Data will be stored for a maximum period of 5 years and then disposed of in a secure manner.

## Abbreviations

CKD: Chronic Kidney Disease
CM: Conservative Management
ESRD: End Stage Renal Disease
HD: Hemodialysis
KDQOL-SF: Kidney Disease Quality of Life short form
MNRH: Mulago National referral Hospital
QoL: Quality of life
